# Sensor-based measurement of critical care nursing workload: Unobtrusive measures of nursing activity complement traditional task and patient level indicators of workload to predict perceived exertion

**DOI:** 10.1371/journal.pone.0204819

**Published:** 2018-10-12

**Authors:** Michael A. Rosen, Aaron S. Dietz, Nam Lee, I-Jeng Wang, Jared Markowitz, Rhonda M. Wyskiel, Ting Yang, Carey E. Priebe, Adam Sapirstein, Ayse P. Gurses, Peter J. Pronovost

**Affiliations:** 1 Armstrong Institute for Patient Safety and Quality, Baltimore, MD, United States of America; 2 Department of Anesthesiology and Critical Care Medicine, The Johns Hopkins University School of Medicine, Baltimore, MD, United States of America; 3 Bloomberg School of Public Health, Department of Health, Policy, and Management; Johns Hopkins University, Baltimore, MD, United States of America; 4 School of Nursing, The Johns Hopkins University, Baltimore, MD, United States of America; 5 The Johns Hopkins University Applied Physics Laboratory, Baltimore, MD, United States of America; 6 The Johns Hopkins Health System, Baltimore, MD, United States of America; 7 Department of Applied Mathematics and Statistics, The Whiting School of Engineering, The Johns Hopkins University, Baltimore, MD, United States of America; 8 Malone Center for Engineering in Healthcare, The Whiting School of Engineering, The Johns Hopkins University, Baltimore, MD, United States of America; University of Illinois at Urbana-Champaign, UNITED STATES

## Abstract

**Objective:**

To establish the validity of sensor-based measures of work processes for predicting perceived mental and physical exertion of critical care nurses.

**Materials and methods:**

Repeated measures mixed-methods study in a surgical intensive care unit. Wearable and environmental sensors captured work process data. Nurses rated their mental (ME) and physical exertion (PE) for each four-hour block, and recorded patient and staffing-level workload factors. Shift was the grouping variable in multilevel modeling where sensor-based measures were used to predict nursing perceptions of exertion.

**Results:**

There were 356 work hours from 89 four-hour shift segments across 35 bedside nursing shifts. In final models, sensor-based data accounted for 73% of between-shift, and 5% of within-shift variance in ME; and 55% of between-shift, and 55% of within-shift variance in PE. Significant predictors of ME were patient room noise (ß = 0.30, *p* < .01), the interaction between time spent and activity levels outside main work areas (ß = 2.24, p < .01), and the interaction between the number of patients on an insulin drip and the burstiness of speaking (ß = 0.19, *p* < .05). Significant predictors of PE were environmental service area noise (ß = 0.18, *p* < .05), and interactions between: entropy and burstiness of physical transitions (ß = 0.22, *p* < .01), time speaking outside main work areas and time at nursing stations (ß = 0.37, p < .001), service area noise and time walking in patient rooms (ß = -0.19, *p* < .05), and average patient load and nursing station speaking volume (ß = 0.30, *p* < .05).

**Discussion:**

Analysis yielded highly predictive models of critical care nursing workload that generated insights into workflow and work design. Future work should focus on tighter connections to psychometric test development methods and expansion to a broader variety of settings and professional roles.

**Conclusions:**

Sensor-based measures are predictive of perceived exertion, and are viable complements to traditional task demand measures of workload.

## Introduction

The increasing workload under which physicians and nurses operate in today’s health care system adversely impacts patient outcomes (i.e., patient experience,[1] healthcare-acquired infections,[2] delays in treatment,[3] postoperative complications, [4] unplanned extubations, [5] and mortality[6,7]), workforce outcomes (i.e., burnout and job-dissatisfaction, [8] as well as turnover and disengagement from or exiting the professions[9–11]), and organizational efficiency and productivity. [12,13] Workload is the level of effort required to complete a task in relation to the resources available to expend on that task. [14,15] When demands exceed available resources, an individual’s performance deteriorates. Despite the importance of workload, there remains a gap in strategies to measure it for health care professionals. Most methods rely on some form of staffing ratio[16,17] that inadequately represents workload. [18] Other workload measurement methods are observation[19] or self-report, [20] which are expensive and burdensome for respondents, respectively. To better understand and manage workload, more dynamic measurement is needed.

Recent advances in low cost, wearable and environmental sensors offer the potential for large scale, unobtrusive measurement of work processes and related constructs. [21–23] Compelling feasibility studies demonstrate the potential utility of sensor data for understanding workforce issues [24,25] and patient data [26,27] but few examples provide rigorous evidence that wearable and environmental sensors can validly measure work processes in vivo. [28]

The objectives of the study were to evaluate whether, after accounting for variance associated with traditional measures of workload, sensor-based measures of work processes could predict significant variance in nurses’ perceived mental and physical exertion while performing demanding tasks in a surgical ICU.

## Materials and methods

### Study design, setting, participants

This prospective repeated measures mixed-methods study was conducted in one surgical ICU at a large urban academic hospital in the Mid-Atlantic United States; study period was July and August, 2014. Eight critical care nurses from the unit were recruited through email and flyer notifications. The study was approved by the Johns Hopkins University School of Medicine Institutional Review Board.

### Sensor-based measurement system

The sensor-based measurement system included wearable and stationary sensor badges equipped with a radiofrequency identification (RFID) 2.4 GHz band (Sociometric Solutions, Inc., Boston, MA)[29] and an infrared sensor (TFDU4300Vishay, Malvern, PA) that captured physical proximity and location. Also, two omnidirectional micro-electrical-mechanical system (MEMS) microphones (SPM0103-NE3, Knowles Electronics, LLC, Itasca, IL) captured features of speech and environmental noise, and a three-axis MEMS accelerometer (ADXL330, Analog Devices, Inc., Norwood, MA) captured body movement and activity. Audio signals were filtered on-board the sensor badge to extract speech features without saving the full signal.

**Fig 1:**
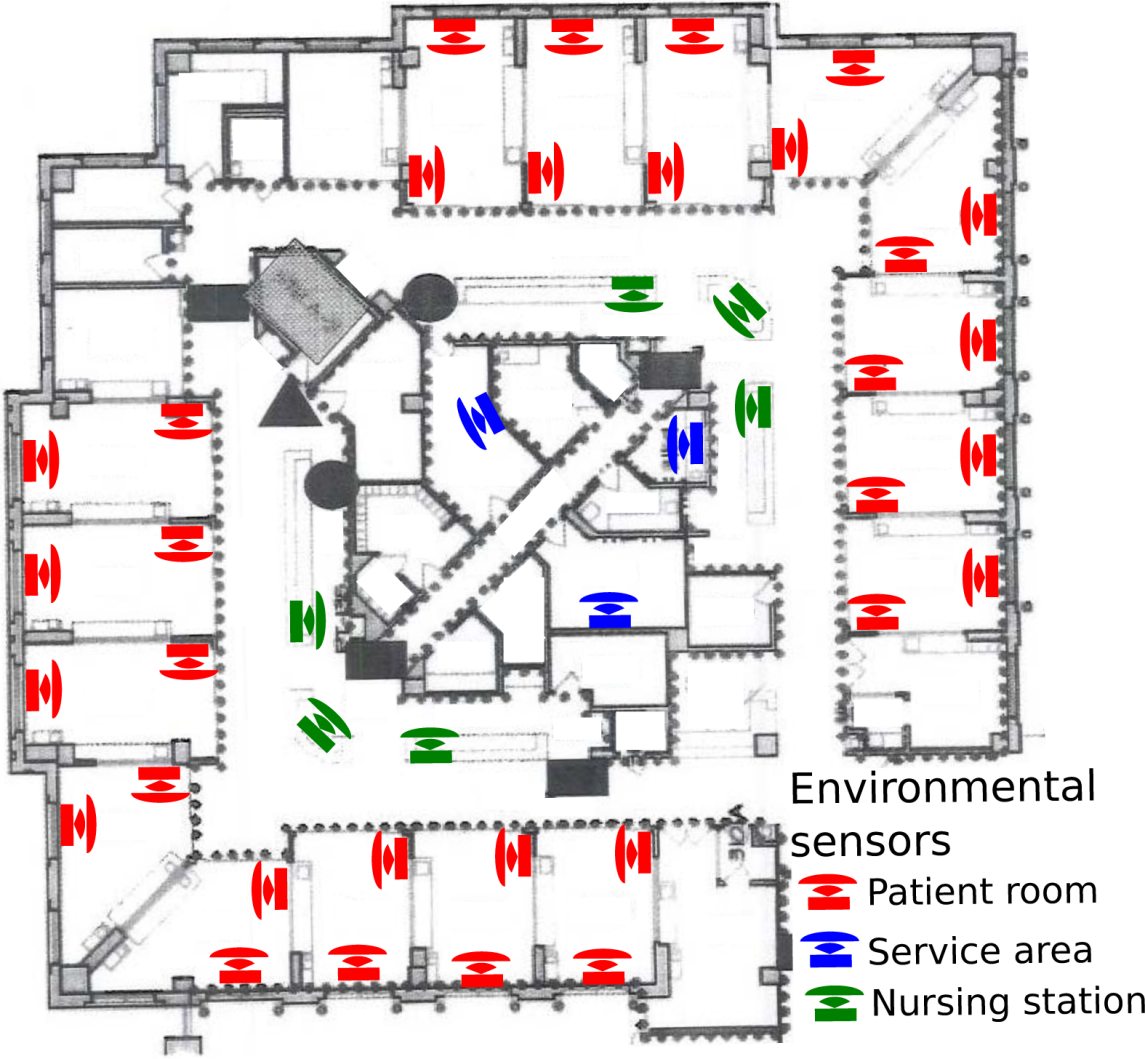
Environmental sensor placements throughout surgical intensive care unit sixteen patient rooms were instrumented with two sensor badges, one immediately inside the room by the computer terminal and the second on the wall opposite the door; two nursing stations were instrumented with three sensor badges; and three services areas (medication, supply, and nutrition) were instrumented with one sensor badge. The service areas were relatively isolated from other work areas and redundant sensors were not needed for accurate localization. Two low occupancy and two isolation rooms on the unit were excluded from this study.

Nurse participants wore a sensor badge and their location was detected through a network of 41 stationary sensor badges placed in 16 of 20 patient rooms, both nursing stations, and three service areas (medication, supply, and nutrition rooms; see Fig 1). A feature engineering process mapped sensor capabilities to nursing work processes using four separate one hour focus group sessions with eight RNs, four hours of observing nurses at work by an experienced human factors researcher, and review of an existing nursing task taxonomy for ICUs.[19] This process resulted in 72 features organized into seven high level categories: location-based (time in location, movement through physical space), accelerometer-based (body movement and activity in location), environmental noise (volume), speaking (time speaking, pitch, volume, burstiness [distribution of activity over time]), posture, walking (time and burstiness of walking), and temperature. All sensor-based measures and definitions are in Supplementary Methods A in S1 File.

### Focus groups and survey instrument

We used nursing focus groups to identify patient and shift level drivers of workload most meaningful in our study unit. Human Factors professionals moderated a total of four sessions, each one hour in duration, with the eight RNs participating in this study. Nursing task demands identified by focus group participants and included in this study cover staffing factors (number of patients, number of patients assigned a sitter, and whether nurse had an assistant), and patient factors (e.g., number of assigned patients requiring specific care interventions; 8 variables). Composite measures of task demand are also reported in Supplementary Methods B in S1 File including descriptions of all variables and measurement definitions.

A brief survey instrument was developed to collect data on staffing and patient task demands, to elicit perceptions of physical exertion (PE) and mental exertion (ME), and to link the sensor data to the completed survey. Perceptions of exertion were measured using the 15-grade Borg Scale for rating PE (scale range, 6 = very, very light to 20 = very, very hard) [30] and a version modified to rate ME. The modified ME scale changed the referent of the survey item from ‘physical’ to ‘mental’ exertion, and the response scale was unchanged. We chose this approach because evidence has established the validity of concurrently measuring mental and physical exertion as related yet distinct sub-dimensions of an overall exertion construct. [31]

### Data collection

At the beginning of each shift, a participating nurse retrieved a sensor badge stored on the unit and recorded the badge number on part one of the brief survey. During the shift, the sensors recorded features of their activity. Nurses rated their perceptions of PE and ME every four hours on the survey and recorded patient and staffing task demands at the end of the shift. Four-hour blocks within a shift were chosen because it corresponded to natural breaks in nursing workflow.

### Data analysis

Data analysis proceeded in two phases: 1) feature selection, and 2) multi-level modeling (MLM). Feature selection was conducted to determine whether any of the 72 sensor-based measures were predictive of nurses’ perceived ME or PE. Elastic net methods, which combine the least absolute shrinkage and selection operator (LASSO) and ridge regression penalties [32,33], were applied to select a parsimonious set of predictors for consideration in MLM. An extension of Elastic net[34] was used to explore all pairwise combinations of predictive features for significant interactions. Feature selection was performed in R (version 3.2)[35] using glmnet (version 2.0–2)[36] and glinternet (version 1.0.0)[37] packages. Elastic net methods do not account for clustering in the data, therefore a more lenient shrinkage penalty was selected so important predictors were not eliminated at this stage. Subsequently, a traditional backwards elimination process was used in MLM with the shift grouping structure in place to further reduce the feature set.

MLM was used to evaluate the predictive validity of sensor-based measures, and conducted with R (version 3.2) using nlme (version 3.1–122)[38] and multilevel (version 2.5)[39] packages. MLM was chosen to account for the non-independence of data collected in four-hour segments within a shift and test cross-level interactions between task demand variables and sensor-based measures. Shift was the grouping variable used to analyze perceptions of ME and PE as dependent variables, sensor-based measures of work processes as Level 1 predictors, and task demand workload variables as Level 2 predictors. All sensor-based measures were grand mean centered prior to analyses. Intraclass correlation coefficients (ICC) measured the proportion of variance between different shifts relative to four-hour segments within the same shift. Model deviance was computed to compare model fit of MLM using an L ratio test. An alpha level of < 0.05 was used for assessing significance. Supplementary Methods C in S1 File provides full detail on data analysis methods.

## Results

Our analysis included 89 four-hour shift segments across 35 bedside nursing day shifts (between 7 AM and 7 PM), totaling 356 work hours of data collected in July and August, 2014. Seventy percent (62/89) were weekday shift segments.

### Feature selection

Elastic net analyses selected 23 variables related to ME from the initial pool of 72 (listed in Supplementary Methods D in S1 File) and 6 interaction terms, as well as 14 variables related to PE and 6 interaction terms. Each of the features retained as either a main effect or interaction term are indicated in Supplementary Methods D in S1 File. Two tuning parameters are used for Elastic net: α which specifies the degree of mixing of penalties from LASSO and ridge regression and λ which controls the degree of shrinkage. Both α and λ can range from 0 to 1. An α of 0 indicates a pure ridge regression penalty, and an α of 1 indicates a pure LASSO. Values in between indicate a proportional mixing of the penalties. For these analyses, α was set at .9 which more heavily weighted the LASSO penalty. A λ value of 0 indicates no shrinkage is performed, and increasing values indicate more severe shrinking of coefficients. For these analyses, the *lambda*.*min* function of glmnet identified lambda values that minimized cross-validation error (for PE: λ = 0.18; and for ME: λ = 0.19).

### Multi-level modeling (MLM)

Tables 1 and 2 detail results of MLM for ME and PE, respectively. Level 1 variables included sensor-based measures as predictors and perceived exertion as dependent variables collected for each four-hour shift segment. Level 1 variables were grouped within shift, and Level 2 variables were task demands associated with that specific shift such as the number of patients cared for and their status level. We detail each step of the MLM process below, followed by a summary of the final ME and PE models.

**Table 1:** Results of multilevel modeling for perceived mental exertion (ME).

	ME_0_: Intercept Only			ME_1_: + Level 1 Predictors			ME_2_: + Level 2 Predictors			ME_3_: + Random Coefficients			ME_4_: + Cross-level Interactions		
**Fixed Components**^a^^,^^b^															
*Predictor*	*ß [S*.*E*.*]*	*df*	*p*	*ß [S*.*E*.*]*	*df*	*p*	*ß [S*.*E*.*]*	*df*	*p*	*ß [S*.*E*.*]*	*df*	*p*	*ß [S*.*E*.*]*	*df*	*p*
1. Intercept	0.02 [0.15]	54	.90	-0.23 [0.18]	50	.21	-0.23 [0.17]	50	.20	-0.10 [0.18]	49	.58	-0.19 [0.17]	48	.26
2. Environmental noise in patient rooms	**-**	**-**	**-**	0.28 [0.12]	50	.02	0.27 [0.12]	50	.02	0.27 [0.10]	49	.008	0.30 [0.10]	48	.003
3. Time outside of main work areas	**-**	**-**	**-**	0.87 [0.67]	50	.20	0.73 [0.66]	50	.27	-0.16 [0.81]	49	.84	0.23 [0.76]	48	.76
4. Activity level outside of main work areas	**-**	**-**	**-**	-0.33 [0.20]	50	.11	-0.36 [0.19]	50	.07	-0.30 [0.18]	49	.09	-0.32 [0.17]	48	.08
5. Time outside of main work areas x Activity level outside of main work areas	**-**	**-**	**-**	2.01 [0.84]	50	.02	2.10 [0.81]	50	.01	2.34 [0.75]	49	.003	2.24 [0.73]	48	.004
6. Burstiness of speaking	**-**	**-**	**-**	-	-		-	-	-	0.08 [0.12]	49	.54	0.03 [0.11]	48	.78
7. Patients on an insulin drip	**-**	**-**	**-**	-	-		0.30* [0.12]	33	.01	0.05 [0.12]	33	.68	0.05 [0.11]	33	.64
8. Patients on an insulin drip x Burstiness of speaking	**-**	**-**	**-**	-	-		**-**	**-**	**-**	-	-	**-**	0.19 [0.07]	48	.01
**Random Components**															
Between-shift variance (σ^2^_e_)	0.64 [0.80]			0.46 [0.68]			0.36 [0.60]			0.22 [0.47]			0.17 [0.41]		
Within-shift / residual variance (σ^2^_u0_)	0.38 [0.62]			0.35 [0.59]			0.35 [0.59]			0.35 [0.59]			0.36 [0.60]		
Slope variation (Burstiness of speaking)										0.08 [0.29]			0.02 [0.15]		
**Variance Modelled**^c^															
Level 1: R^2^_Within Shift_				0.08			0.05			0.05			0.05		
Level 2: R^2^_Between Shift_				0.28			0.44			0.66			0.73		
Ran. Coef.: R^2^_slope: Burstiness of speaking_										0			.75		
**Model Fit**															
Deviance (-2 * Log likelihood ratio)^d^	224.2			210.00			203.40			195.47			190.43		
L. Ratio	*χ*^2^(1) = 27.30, *p* < .001			*χ*^2^(4) = 14.27, *p* < .01			*χ*^2^(1) = 6.60, *p* < .05			*χ*^2^(3) = 7.93, *p* = .05			*χ*^2^(1) = 5.04, *p* < .05		

^a^There were 89 shift segments nested within 35 shifts.

^b^Terms 2–4, and 6 are Level 1 main effects; Term 5 is a Level 1 interaction; Term 7 is a Level 2 main effect; term 8 is a cross-level interaction.

^c^Proportion of variance explained for Level 1 (within shifts) and Level 2 (between shifts) were calculated relative to ME_0_, the model with only a grouping variable; Proportion of slope variation explained was calculated relative to ME_3_, the model with random coefficients, but no cross-level interactions.

^d^ Reduction in model deviance was tested as follows: ME_0_ contained only the shift grouping variable and was tested against a model without the grouping variable; ME_1_ included Level 1 sensor-based measures and was tested against ME_0_; ME_2_ included Level 2 task demand fixed effects and was compared to ME_1_; ME_3_ included random coefficients for Level 1 sensor-based measures and was evaluated relative to ME_2_, the model without random coefficients; ME_4_ included cross level interactions between Level 1 and Level 2 predictors and was evaluated relative to ME_3_.

**Table 2:** Results of multilevel modeling for perceived physical exertion (PE).

	PE_0_: Intercept Only			PE_1_: + Level 1 Predictors			PE_2_: + Level 2 Predictors^e^			PE_3_: + Random Coefficients			PE_4_: + Cross-level Interactions		
**Fixed Components**^a^^,^^b^															
*Predictor*	*ß [S*.*E*.*]*	*df*	*p*	*ß [S*.*E*.*]*	*df*	*p*	*ß [S*.*E*.*]*	*df*	*p*	*ß [S*.*E*.*]*	*df*	*p*	*ß [S*.*E*.*]*	*df*	*p*
1. Intercept	-0.01 [0.14]	54	.97	-0.01 [0.11]	42	.92	-	-	-	-0.01 [0.11]	43	.9	-0.04 [0.11]	43	.70
2. Entropy of transitions				0.15 [0.10]	42	.11	-	-	-	0.14 [0.09]	43	.11	0.15 [0.08]	43	.06
3. Burstiness of transitions				-0.07 [0.12]	42	.57	-	-	-	-0.6 [0.10]	43	.58	-0.03 [0.09]	43	.76
4. Time speaking outside of main work areas				-0.02 [0.10]	42	.84	-	-	-	0.03 [0.09]	43	.71	0.03 [0.09]	43	.78
5. Volume while speaking at nursing stations				0.07 [0.09]	42	.44	-	-	-	0.02 [0.14]	43	.87	0.05 [0.13]	43	.68
6. Time at nursing stations				0.16 [0.10]	42	.14	-	-	-	0.16 [0.10]	43	.10	0.17 [0.09]	43	.08
7. Environmental noise in service areas				0.19 [0.09]	42	.04	-	-	-	0.19 [0.08]	43	.03	0.18 [0.08]	43	.03
8. Time walking in patient rooms				0.09 [0.10]	42	.36	-	-	-	0.12 [0.09]	43	.18	0.12 [0.09]	43	.18
9. Temperature in service areas				-0.04 [0.09]	42	.62	-	-	-	-0.04 [0.09]	43	.65		-	
10. Entropy of transitions x Burstiness of transitions				0.19 [0.09]	42	.04	-	-	-	0.23 [0.07]	43	.004	0.22 [0.07]	43	.003
11. Time speaking outside of main work areas x Time at nursing stations				0.38 [0.11]	42	< .001	-	-	-	0.38 [0.10]	43	< .001	0.37 [0.09]	43	< .001
12. Environmental noise in service areas x Time walking in patient rooms				-0.23 [0.10]	42	.02	-	-	-	-0.21 [0.08]	43	.01	-0.19 [0.08]	43	.02
13. Volume while speaking at nursing stations x Temperature in service areas				-0.14 [0.07]	42	.04	-	-	-	-0.02 [0.08]	43	.86	-	-	
14. Average patient load													-0.05 [0.11]	33	.68
15. Average patient load x Volume while speaking at nursing stations													0.30 [0.13]	43	.02
**Random Components**															
Between shift variance (σ^2^_e_)	0.55 [0.74]			0.19 [0.45]			-	-		0.26 [0.51]			0.25 [0.50]		
Within shift / residual variance (σ^2^_u0_)	0.42 [0.65]			0.32 [0.56]			-	-		0.16 [0.40]			0.20 [0.45]		
Slope variation (Volume while speaking at nursing stations)										0.27 [0.52]			0.16 [0.40]		
**Variance Modelled**^c^															
Level 1: R^2^_Within Shift_				0.24			-	-		0.62			0.52		
Level 2: R^2^_Between Shift_				0.65			-	-		0.53			0.55		
Ran. Coef.: R^2^_Slope: Volume spkng at RN statns_										0			0.41		
**Model Fit**															
Deviance (-2 * Log likelihood ratio)^d^	226.59			183.34						174.25			168.55		
L. Ratio	*χ*^2^(1) = 24.98, *p* < .001			*χ*^2^(12) = 43.25, *p* < .001						*χ*^2^(2) = 9.08, *p* < .05			f		

^a^There were 89 shift segments nested within 35 shifts.

^b^ Terms 2–9 are Level 1 main effects; Terms 10–13 are level 1 interactions; Term 14 is a Level 2 main effect; Term 15 is a cross-level interaction.

^c^Proportion of variance explained for Level 1 (within shifts) and Level 2 (between shifts) were calculated relative to PE_0_, th model with only a grouping variable; Proportion of slope variation explained was calculated relative to PE_3_, the model with random coefficients, but no cross-level interactions.

^d^Reduction in model deviance was tested as follows: PE_0_ contained only the shift grouping variable and was tested against a model without the grouping variable; PE_1_ included Level 1 sensor-based measures and was tested against PE_0_; PE_2_ included Level 2 task demand fixed effects and was compared to PE_1_; PE_3_ included random coefficients for Level 1 sensor-based measures and was evaluated relative to PE_2_, the model without random coefficients; PE_4_ included cross level interactions between Level 1 and Level 2 predictors and was evaluated relative to PE_3_.

^e^No Level 2 task demand variables were retained as significant in PE_2_. Therefore, PE_2_ = PE_1_.

^f^For PE_4_, model deviance was reduced, but model complexity (due to the loss of degrees of freedom by the inclusion of nonsignificant main effects for significant interactions) precluded significance testing

ICC values supported the use of shift as the grouping structure for ME (ICC = 0.63) and PE (ICC = 0.57), indicating that 63% of total variance in ME and 57% in PE occurred between shifts. Group mean reliability exceeded the standard of 0.7 for both ME (0.81) and PE (0.76). Both ME_0_ (*χ*^2^(1) = 27.30, *p* < .001) and PE_0_ (*χ*^2^(1) = 24.98, *p* < .001) had significantly better fit than models without the shift grouping variable.

Models ME_1_ and PE_1_ added Level 1 sensor-based predictors. To generate ME_1_ and PE_1_, all features retained from Elastic net analysis (i.e., 23 main effects and six interaction terms for ME; 14 main effects and 6 interaction terms for PE) were added to the respective ME_0_ or PE_0_ model which included the shift grouping structure, and a traditional backward elimination process was performed. ME_1_ and PE_1_, as detailed in Tables 1 and 2 respectively, represent the end of the backward elimination feature reduction process. This process produced a model for ME with one significant main effect term and one significant interaction term, accounting for 28% of the between and 8% of within shift variance. Model PE_1_ was reduced to four interaction terms (Table 2, predictors 10 to 13), accounting for 65% of between shift and 24% of within shift variance. Models ME_1_ and PE_1_ were significantly better fitting models compared to ME_0_ (*χ*^2^(4) = 14.27, *p* = .007) and PE_0_ (*χ*^2^(12) = 43.25, *p* < .001), respectively.

Models ME_2_ and PE_2_ added task demands documented by nurses working that shift (Level 2). One task demand, number of patients on an insulin drip, was a significant predictor of ME, producing a model that accounted for 44% of between-shift and 5% of within-shift variances. Model ME_2_ exhibited a significantly better fit compared to ME_1_ (*χ*^2^(1) = 6.60, *p* = .01). No task demand predictors were retained for PE. Therefore, PE_2_ was equivalent to PE_1_.

One significant random coefficient term was retained in Models ME_3_ (burstiness of speaking) and PE_3_ (volume while speaking at nursing stations), producing significantly better fitting models compared to ME_2_ (*χ*^2^(3) = 7.93, *p* = .05) and PE_2_ (*χ*^2^(2) = 9.08, *p* < .05), respectively. Model ME_3_ accounted for 66% of between shift and 5% of within shift variances. Model PE_3_ accounted for 53% of between-shift, and 62% of within-shift variances. In Model PE_3_, a previously significant main effect term (temperature in service areas) and interaction term (volume while speaking at nursing stations by temperature in service areas) became non-significant and were excluded from further analysis.

**Fig 2:**
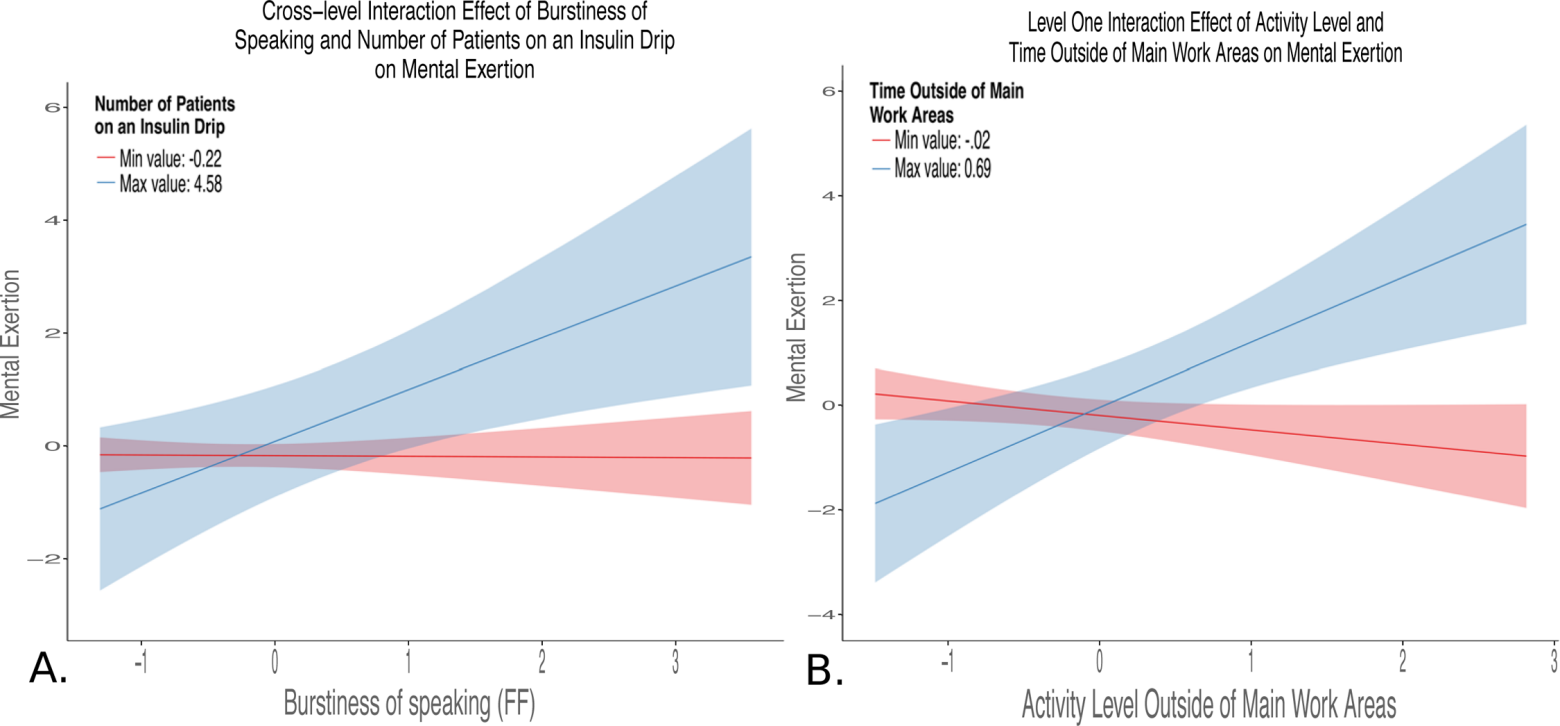
Interaction terms for final reduced mental exertion model. Blue lines represent the maximum value (upper bound), and red lines indicate the minimum value (lower bound) for the Level 2 variable to illustrate the interaction. The shaded areas around each line indicates the 95% confidence region surrounding the upper and lower bounds moderator variable. **Panel A:** Cross-level interaction between burstiness of speaking (Level 1 sensor-based measure) and number of patients on an insulin drip (task workload factor) on mental exertion. This illustrates that the task demand of patients on an insulin drip positively moderates the relationship between the burstiness of speaking and mental exertion, such that high levels of burstiness of speaking become more predictive of high levels of mental exertion when caring for patients on an insulin drip. **Panel B:** Level 1 interaction illustrated that high levels of activity outside of main work areas and longer time outside of main work areas were predictive of high levels of mental exertion.

**Fig 3:**
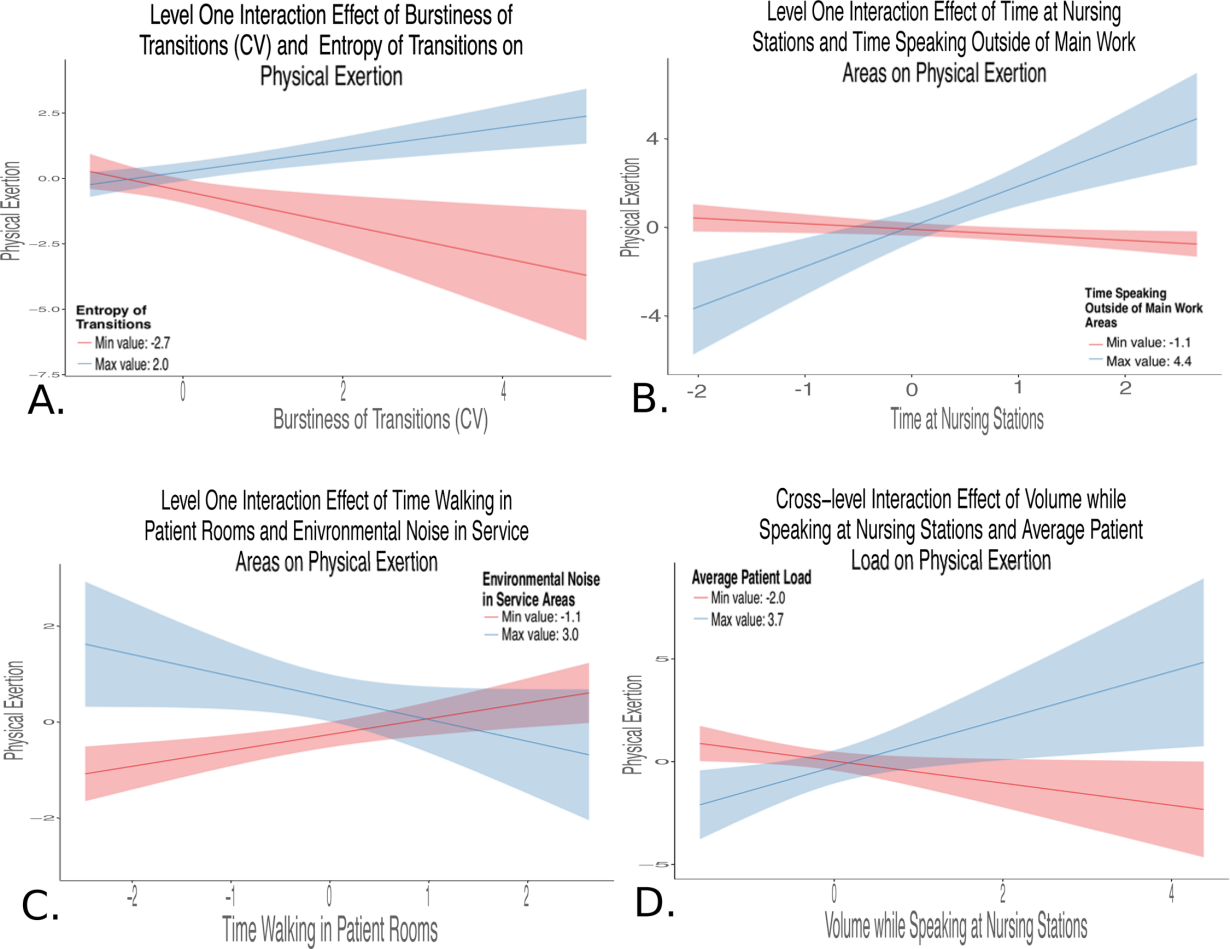
Interactions for final reduced physical exertion model blue lines represent the maximum value (upper bound), and red lines indicate the minimum value (lower bound) of the moderator variable to illustrate the interaction between the independent variable and moderator. The shaded areas around each line indicate the 95% confidence region surrounding the upper and lower bounds moderator variable. **Panel A:** This positive interaction between two sensor-based measures (Level 1) indicates that work shifts with physical transition events that are both highly unstructured (high entropy) and bursty (high clumping together of transition events in time) are more physically exerting. **Panel B:** Level 1 interaction illustrates the positive conditional effects in which higher levels of time at nursing station and higher levels of time speaking outside of main work areas are associated with higher levels of physical exertion. **Panel C:** Level 1 interaction illustrates a negative effect in which less time walking in the patient rooms and high levels of environmental noise in service areas were associated with higher physical exertion. **Panel D:** Cross-level positive interaction between volume of speaking at the nursing station (Level 1) and average patient load (task workload factor) indicated that a general vocal stress indicator (speaking volume) is only significantly associated with physical exertion when localized to the nursing station and when caring for more complex patients.

One significant cross-level interaction was retained in Models ME_4_ and PE_4_. The final model ME, ME_4_, included a significant and positive cross-level interaction between a Level 2 task demand variable, number of patients on an insulin drip, and a Level 1 sensor-based measure, burstiness of speaking (ß = 0.19, *p* < .05) as well as the interaction term between two Level 1 sensor-based measures (time spent and activity levels outside of main work, ß = 2.24, *p* < .01) and a Level 1 main effect term (environmental noise in patient rooms, ß = 0.30, *p* < .01). We defined the main patient care or work areas as patient rooms, nursing stations, and service areas. Areas outside of these main patient care areas included unit halls, locker room, and conference or break room areas. Main work areas were instrumented in this study, and areas in the unit outside of this were not.” This model accounted for 73% of between-shift and 5% of within-shift variances, and for 75% of the variation in slopes between burstiness of speaking and mental exertion across shifts. The final model for PE, PE_4_, included a significant and positive cross-level interaction between a Level 2 task demand variable, average patient load, and a Level 1 sensor-based measure, volume while speaking at the nurses’ station (ß = 0.30, *p* < .05) as well as three interaction terms between Level 1 sensor-based measures (entropy by burstiness of physical transitions, ß = 0.22, *p* < .01; time speaking outside of main work areas by time at nursing stations, ß = 0.37, *p* < .001; environmental noise in service areas by time walking in patient rooms, ß = -0.19, *p* < .05) and one Level 1 main effect (environmental noise in service areas, ß = 0.18, *p* < .05). This model accounted for 55% of between-shift and 55% of within-shift variances, and for 41% of variation in slopes between volume while speaking at the nurses’ station and physical exertion across shifts. Both Models ME_4_ and PE_4_ had large decreases in model deviance. This decrease was significant for Model ME_4_ (*χ*^2^(1) = 5.04, *p* < .05), but model complexity (degrees of freedom lost due to including non-significant main effect terms for multiple interaction terms) precluded significance testing for Model PE_4_. Significant Level 1 and cross-level interactions for final reduced models are depicted in Fig 2A and 2B (ME_4_) and Fig 3A through 3D (PE_4_). The interaction plots detailed in Figs 2 and 3 were generated with the r package *sjPlot* [40]. Variables were centered by subtracting the mean value of that variable, and then scaled by dividing values by the standard deviation. Each plot was constructed by plotting the relationship between two of the three interaction terms while holding the third moderator variable constant at the upper (maximum value depicted in blue) and lower (minimum value depicted in red) bounds. Fig 2A illustrates a cross-level interaction where burstiness of speaking, a Level 1 predictor, becomes a stronger predictor of mental exertion with increasing numbers of patients on an insulin drip, a Level 2 predictor. Fig 2B shows the positive interaction between two Level 1 predictors, activity levels and time spent outside of main work areas, on mental exertion. Fig 3A illustrates that work shifts with higher levels of entropy and burstiness of transitions are more physically exerting. Fig 3B shows that shifts with higher levels of time at nursing stations and time spent speaking outside of main work areas were more physically exerting. Fig 3C illustrates a negative interaction between time spent walking in patient rooms and noise in service areas predicted physical exertion. Fig 3D shows a cross-level interaction where volume while speaking at the nurses’ station became a stronger predictor of physical exertion with increasing levels of average patient load.

## Discussion

In the final reduced models for mental and physical exertion, sensor-based measures of work processes accounted for large proportions of unique variance above and beyond task demand variables typically used for evaluating workload (i.e., task demands derived from patient and shift level factors). These findings support the further development of these technologies for workforce management issues in healthcare. The significant cross-level interactions our models are consistent with existing multi-level frameworks of nursing workload[41] in that relationships between different work processes and perceived exertion changed based on higher level task demand workload factors.

### Main study findings

The final model for ME included noise in patient rooms, an interaction between time spent and activity levels outside the main areas, and an interaction between number of patients on an insulin drip and burstiness of speaking. Environmental noise is a well-documented stressor with a positive relationship with perceptions of workload.[42,43]

As illustrated in Fig 2B, the positive interaction between time and activity levels outside of main work areas indicated that the more time spent away from patient rooms, nursing stations, or service areas when activity levels were high outside of these areas the higher the nurse’s mental exertion. High activity levels outside the main patient care areas could mean the nurse was searching for team member support or supplies, while low levels of activity could indicate downtime. For example, high activity in non-work areas could involve walking up and down the unit halls to seek assistance, and low activity in non-main work areas could involve socializing in a break room.

As illustrated in Fig 2A, the positive interaction between number of patients on an insulin drip and the burstiness of speaking indicated that certain sensor-based measures were predictive of mental workload when caring for patients requiring specific care interventions. The burstiness of speaking is a measure of the temporal distribution of time spent speaking. Higher levels of burstiness of speaking means speaking is more clumped together in time with periods of relatively intensity and sparseness, and lower levels mean a more even distribution of speaking over time. Insulin infusion protocols improve outcomes for ICU patients,[44] but the nursing workload associated with these complex protocols is known to be high.[45] For each patient on an insulin drip, a nurse must assess blood sugar levels, make complex calculations, enter changes into the infusion pump, document all information, and find a second nurse to independently double check the completeness of the steps. Fig 2A illustrates that this bursty social dynamic, potentially an indicator of interruptions[46] or challenges in finding an available nurse to perform the independent double check, was only significantly associated with mental exertion in the context of managing patients on insulin drips.

The final reduced model for physical exertion had one significant main effect and positive as well as negative interactions. First, environmental noise within services areas was the main predictor for physical exertion. This potentially indicates congestion in these areas contributing to perceptions of physical effort.

Second, as shown in Fig 3A, the interaction between entropy of transitions and burstiness of transitions pertained to patterns of movement through physical space. Entropy of transitions was calculated using the Shannon entropy of the time series of physical locations. Higher levels of entropy indicated less predictability in the sequence of transitions. The burstiness of transitions characterized the temporal variation of movement events from one physical space to another. Higher levels of burstiness indicated more clumping in time of movement between physical areas. Shift segments where physical transitions were both unstructured and clumped in time were more physically exerting.

Third, as illustrated in Fig 3B, the positive interaction between high levels of time speaking outside of the main work areas and high levels of time spent at nursing stations could indicate care of more complex patients, requiring more documentation and coordination of activities, thereby compressing physical activity in the patient rooms into less time.

A fourth and also challenging interaction to interpret was the negative interaction between environmental noise in service areas and time walking in patient rooms, as shown in Fig 3C. This relationship could indicate that a busier service area (more congested and noisier) and more physical activity in the patient room combined to impact physical workload. These are two areas that require the most physical activity from nurses (e.g., patient handling and procedures in the patient room; moving and lifting supplies in service areas).

Fifth, as illustrated in Fig 3D, the interaction between average patient load and volume while speaking at the nursing stations indicated increased strength in the relationship between speaking volume at the nursing station and physical exertion in shifts where patients required more care interventions. Speaking intensity or volume is a feature of speech commonly associated with stress.[47] In our study, volume was only predictive of perceived physical exertion when localized to the nursing station and related to the level of monitoring and intensity of task demands made on the nurse.

### Implications for future research and practice

This study demonstrated the predictive validity of sensor-based measures. Some features were clearly meaningful, while the interpretation of others was more challenging. A tighter integration with the existing psychometric test development processes is needed to help ensure the content of these sensor-based systems are indicative of the construct purported to be measured. With a refined system, sensor-based measures could be used to guide more fine grained workflow analyses[48] to identify recurrent trends and target these areas for further investigation and improvement efforts, or as real time feedback to help staff on the unit self-regulate and balance workload.[49] Projections indicate severe shortages in nurse[50] and physician[51] workforces for decades to come. Identifying mechanisms to improve productivity and retention could have substantial savings. By better understanding workload in real time, managers can provide lateral support to reduce workload and ultimately create a safer and more productive work environment.

### Limitations

This study was conducted in one surgical critical care unit in an academic medical center. Larger datasets collected across multiple critical care units in different facilities, including a wider range of task demands and clinical roles will be required to establish the generalizability of sensor-based measurement features across settings and personnel. For example, burstiness of speaking was related to mental exertion only when managing patients on insulin drips. This dynamic could be indicative of higher levels of workload in other situations, but this study was underpowered to detect the effect. While this study drew from existing models of nursing workload, qualitative focus groups, and observational methods, it remained largely exploratory. Advances in integrating sensor-based measurement within the psychometric test development framework will be necessary to develop more prospective measure development and validation. In contrast to traditional assessment methods (e.g., self-report and observation) with established best practices and methods, these types of wearable and environmental sensors have generally unknown error structures [52] but systematic device related variance has been demonstrated in other research for sensors like those used in this study [53]. Additionally, detecting naturalistic speech and isolating it to the sensor wearer (vs. others speaking in the area) may be particularly difficult and potentially error prone. We did not formally assess the reliability of all sensor features used in analyses reported here.

## Conclusion

Sensor-based measurement systems are valuable tools for understanding performance in complex socio-technical systems. These methods have the potential to enhance patient safety, improve productivity and reduce burnout among nurses. This approach may be applied to physicians and other health care workers and extended to other types of organizational performance, such as coordination and teamwork which are known drivers of safety and quality yet difficult to measure on a large scale with currently available methods.

## Supporting information

S1 File Supplementary methods for this study. Supplementary Methods A. Supplementary Methods B. Supplementary Methods C. Supplementary Methods D.
(DOCX)

S2 File Data supplement. (CSV)
