# Supplementary material for: Sensor-based measurement of critical care nursing workload: Unobtrusive measures of nursing activity complement traditional task and patient level indicators of workload to predict perceived exertion
Source: PLoS One. 2018 Oct 12;13(10):e0204819. doi: 10.1371/journal.pone.0204819 (PMC6193584; doi:10.1371/journal.pone.0204819)
Supplement: S1 File — Supplementary Methods A. Supplementary Methods B. Supplementary Methods C. Supplementary Methods D. (DOCX) [file pone.0204819.s001.docx]

Online Materials:

Supplementary Methods A: Unobtrusive sensor-based measures of critical care nursing work processes

Supplementary Methods B: Nursing Task Demand Workload Variables

Supplementary Methods C: Multi-level Modeling Methods

Supplementary Methods D: Data Dictionary

**Supplementary Methods A: Unobtrusive sensor-based measures of critical care nursing work processes**

A total of 72 features were extracted from sensor badge data. These measures are grouped into seven general categories: location-based measures, accelerometer-based measures, environmental noise, speaking, posture, walking and temperature.

*Environmental Deployment of Sensors*

Multiple configurations of environmental sensor placements were evaluated in pilot testing. In patient rooms, the combination of one sensor on the wall opposite the door, and one sensor at the nurse’s computer terminal immediately inside the patient room were chosen as the best tradeoff between data reliability (90% accuracy in classifying physical location as determined by an observer) and practical constraints (i.e., the sensors needed to be near electrical outlets, limited number of sensors to deploy). Four of a total 20 patient rooms on the unit were not included in the study. Two isolation rooms were excluded due to challenges in placing and removing sensors; and two low occupancy rooms used for surge capacity were excluded. Ultimately, 41 environmental sensors were deployed: two sensors in each of 16 patient rooms, three at each of two nursing stations, and one sensor in each of three service areas (i.e., medication, supply, and nutrition rooms).

**LOCATION-BASED MEASURES (L)**

Participant location was inferred using a combination of infrared (IR) and RFID sensors. Environmental sensors were deployed as depicted in Figure 1. Both IR and RFID measures were used to localize nurses to specific rooms or nursing stations. The IR sensors in the badge system used in this study have high specificity (i.e., few false positives; e.g., if a person was detected by an IR sensor in a given location, they were almost certainly in that location) and low sensitivity (i.e., many false negatives; e.g., if an IR sensor did not detect a person in a location, it can not be ruled out that that person was in that location). The RFID sensors have the opposite profile with high sensitivity (i.e., few false negatives) and low specificity (i.e., many false positives). These different profiles result from the relatively narrow detection field for the IR sensors, and the inherently noisy RFID detections as radio waves deflect from and are absorbed by different surfaces. The localization strategy accounted for these detection profiles in the following way.

For each 20 second time window during a shift, the total number of IR and RFID detections were summated for each individual nurse and location (individual sensors for the three service areas, pairs of sensors for each of the 16 patient rooms, and three sensors for each of the two nursing stations). If the total IR detections were non zero, the nurse was categorized as being in the location with the largest number of IR detections. If total IR detections were zero (which was most common), the nurse was categorized as being in the location with the largest number of RFID detections. If both total IR and RFID detections were zero, the nurse was categorized as being ‘outside of main work areas’. There were a total of 21 unique physical locations (16 patient rooms, 2 nursing stations, 3 service areas) as well as a category for ‘outside of main work areas’, yielding a total of 22 categories for physical location. In pilot studies this approach reached 90% accuracy in localizing nurses in an ICU.

**L1. Time in location category:** Measures of time in types of locations were created by aggregating the 22 specific location categories into higher level categories: patient rooms, nursing stations, service areas, and outside of main work areas. Variables for both total and average time in each of these four higher level location categories were created.

- The **average time in patient rooms** = The total number of 20 second time windows where a nurse was categorized as being in one of the 16 patient rooms divided by the total number of 20 second time windows in the shift segment
- The **average time at nursing stations** = The total number of 20 second time windows where a nurse was categorized as being at one of the two nursing stations divided by the total number of 20 second time windows in the shift segment
- The **average time in service areas** = The total number of 20 second time windows where a nurse was categorized as being in one of the three service areas divided by the total number of 20 second time windows in the shift segment
- The **average time outside of main work areas** = The total number of 20 second time windows were a nurse was categorized as being outside of main work areas divided by the total number of 20 second time windows in the shift segment
- The **total time in patient rooms** = The total number of 20 second time windows where a nurse was categorized as being in one of the 16 patient rooms
- The **total time at nursing stations** = The total number of 20 second time windows where a nurse was categorized as being at one of the two nursing stations
- The **total time in service areas** = The total number of 20 second time windows where a nurse was categorized as being in one of the three service areas
- The **total time outside of main work areas** = The total number of 20 second time windows were a nurse was categorized as being outside of main work areas

**L2. Movement through physical space:** Four measures were used to characterize movement through physical space. For each of these measures, the 22 specific location categories were used, and not the four higher level location categories. The time series of 22 specific location categories were transformed into a time series of transition events by comparing each location category to the one preceding it. If the two locations were the same, no transition event occurred (coded as a 0 in a new time series); if the two locations were different, a transition event did occur (coded as a 1 in a new time series). The number of transitions and burstiness measures are based on the time series of transition events, and the entropy of transitions is based on the original time series of 22 specific location categories.

- The **number of transitions** = The total number of times a nurse transitioned from one of the 22 location categories to another during a shift segment.
- The **entropy of transitions** = The Shannon entropy of the time series of locations (characterized using the 22 specific location categories) for an entire shift segment
- The **burstiness of transitions (FF)** = The mean Fano Factor for transition events calculated over 10 minute windows throughout the shift segment. Specifically, the variance in transition events divided by the mean of transition events for a 10 minute window was calculated and averaged for a shift segment. This represented 30 opportunities for a transition to occur (i.e., each 20 second interval for which a nurse was categorized into a location).
- The **burstiness of transitions (CV)** = The mean coefficient of variation for transition events calculated over 10 minute windows throughout the shift segment. Specifically, the standard deviation in transition events divided by the mean of transition events for a 10 minute window was calculated and averaged for a shift segment.

**ACCELEROMETER-BASED MEASURES (A)**

**A1. Overall body movement and activity:** Overall activity data were extracted from the magnitude of energy across the three-axis accelerometer using the Sociometric Solutions, Inc. software. Specifically, two related measures were generated from this data: body movement and activity.

- The **overall body movement** = The magnitude of energy across the three-axis accelerometer, sampled in 20 second intervals and averaged across an entire shift segment.
- The **overall activity** = The absolute value of the first derivative of the magnitude of energy across the three-axis accelerometer, sampled in 20 second intervals and averaged across an entire shift segment.

**A2. Accelerometer-based measures by location:** Body movement and activity measures were combined with location-based measures to create average measures of each accelerometer measure in each of the four high level location categories.

- The **body movement in patient rooms** = Body movement while participant is located in a patient room averaged across a shift segment
- The **activity in patient rooms** = Activity while participant is located in a patient room averaged across a shift segment
- The **body movement at nursing stations** = Body movement while participant is located at nursing stations averaged across a shift segment
- The **activity at nursing stations** = Activity while participant is located at nursing stations averaged across a shift segment
- The **body movement in service areas** = Body movement while participant is located in service areas averaged across a shift segment
- The **activity in service areas** = Activity while participant is located in service areas averaged across a shift segment
- The **body movement outside main work areas** = Body movement while participant is located outside of the main work areas (patient rooms, nursing stations, and service areas) averaged across a shift segment
- The **activity outside main work areas** = Activity while participant is located outside of the main work areas (patient rooms, nursing stations, and service areas) averaged across a shift segment

**ENVIRONMENTAL NOISE (E)**

Environmental noise is a well-documented source of stress.[1] Environmental noise was operationalized in this measurement system as volume levels across time regardless of whether or not the individual wearing the badge was detected as speaking or not.

**E1. Environmental Noise overall:** Two overall environmental noise measures were used, one from each of the two onboard microphones. These two measures were highly correlated.

- The **overall environmental noise (FV)** = the average volume level of the front facing microphone across all locations for a shift segment
- The **overall environmental noise (BV)** = the average volume level of the back facing microphone across all locations for a shift segment.

**E2. Environmental Noise by location:** Environmental noise overall measures were combined with location-based measures to create average measures of each environmental noise overall measure in each of the four high level location categories.

- The **environmental noise (FV) in patient rooms** = the average volume level of the front facing microphone while participant is located in a patient room for a shift segment
- The **environmental noise (BV) in patient rooms** = the average volume level of the back facing microphone while participant is located in a patient room for a shift segment
- The **environmental noise (FV) at nursing stations** = the average volume level of the front facing microphone while participant is located at a nursing station for a shift segment
- The **environmental noise (BV) at nursing stations** = the average volume level of the back facing microphone while participant is located at a nursing station for a shift segment
- The **environmental noise (FV) in service areas** = the average volume level of the front facing microphone while participant is located in a service area for a shift segment
- The **environmental noise (BV) in service areas** = the average volume level of the back facing microphone while participant is located in a service area for a shift segment
- The **environmental noise (FV) outside of main work areas** = the average volume level of the front facing microphone while participant is located outside of main work areas for a shift segment
- The **environmental noise (BV) outside of main work areas** = the average volume level of the back facing microphone while participant is located in outside of main work areas for a shift segment

**SPEAKING (S)**

Time spent speaking was extracted from audio data by the Sociometric Solutions, Inc. software. Speech is detected by segmenting audio data and classifying based on dominant frequencies. Specifically, “[t]he sociometric badges have an analogue band-pass filter bank that divides the speech frequency spectrum (85, 4,000) Hz into four frequency bands: f_1_ from 85 to 222 Hz, f_2_ from 222 to 583 Hz, f_3_ from 583 to 1,527 Hz, and f_4_ from 1,527 to 4,000 Hz”.[ 2, p. 82] Audio data that fall within the first range (85 Hz to 222 Hz) are classified as speaking segments because they represent the typical speaking energy of adult males and females. The sociometric badges contain two omni-directional microphones, one facing forward (away from the wearer) and one facing upward (toward the speaker’s face). The housing of the microphones helps to focus the receptive field of each microphone in one of the two directions stated above (i.e., the microphone is inside the badge, with a small hole limiting acoustic signals reaching the microphones). In this way, signals from the upward facing microphone can be used to isolate speech and improve voice activity detection. For each shift, a participant was classified as speaking or not for each second of the shift.

**S1. Time speaking overall:** Two summary measures for speaking were included in the analysis.

- The **total time speaking** = the total number of seconds across an entire shift segment where a participant was classified as speaking.
- The **mean time speaking** = the total number of seconds spent speaking per time window (20 seconds in this analysis) divided by the total number of time windows in the shift segment.

**S2. Time speaking by location:** The two overall time speaking measures were crossed by the four high level categories of locations (patient room, nursing station, service areas, outside main work areas) to generate eight measures of time speaking by location.

- The **total time speaking in patient room** = the total number of seconds where a participant was categorized as speaking while that participant is located in a patient room for a shift segment
- The **mean time speaking in patient room** = the average number of seconds per 20 second time window where a participant was categorized as speaking while that participant is located in a patient room for a shift segment
- The **total time speaking at nursing station** = the total number of seconds where a participant was categorized as speaking while that participant is located at a nursing station for a shift segment
- The **mean time speaking at nursing station** = the average number of seconds per 20 second time window where a participant was categorized as speaking while that participant is located at a nursing station for a shift segment
- The **total time speaking in service areas** = the total number of seconds where a participant was categorized as speaking while that participant is located in a service area for a shift segment
- The **mean time speaking in service areas** = the average number of seconds per 20 second time window where a participant was categorized as speaking while that participant is located in a service area for a shift segment
- The **total time speaking outside main work areas** = the total number of seconds where a participant was categorized as speaking while that participant is located outside of main work areas for a shift segment
- The **mean time speaking outside main work areas** = the average number of seconds per 20 second time window where a participant was categorized as speaking while that participant is located outside of main work areas for a shift segment

**S3. Burstiness of speaking:** Two measures of ‘burstiness’ were used to characterize the temporal distribution of speaking activity. These were calculated using the same methods described for burstiness of transitions above.

- The **burstiness of speaking (FF)** = The Fano Factor of speaking
- The **burstiness of speaking (CV)** = The coefficient of variation for speaking

**S4. Pitch while speaking (overall):** Pitch while speaking represents the value along the speech frequency spectrum. The overall pitch while speaking is operationalized as the frequency of sound for one second intervals where participants have been identified as speaking for a shift segment. Pitch was standardized within shift so that the pitch measure controlled for individual differences in baseline pitch of the speaker.

- The **overall pitch while speaking** = The pitch level for each second a participant was categorized as speaking averaged across a shift segment

**S5. Pitch while speaking by location:** The overall pitch while speaking variable was crossed with the four high level location categories to create four pitch by location measures.

- The **pitch while speaking in patient rooms** = the average pitch while speaking when a participant is located in a patient room for a shift segment
- The **pitch while speaking at nursing stations** = the average pitch while speaking when a participant is located at a nursing station for a shift segment
- The **pitch while speaking in service areas** = the average pitch while speaking when a participant is located in a service area for a shift segment
- The **pitch while speaking outside main work areas** = the average pitch while speaking when a participant is located outside of main work areas for a shift segment

**S6. Volume while speaking (overall):** Volume data were extracted from Sociometric Solutions, Inc. software. Specifically, for each second the participant was identified as speaking, the volume level of the back facing microphone was sampled. Volume was standardized within shift so that the volume while speaking measure controlled for individual differences in baseline volume of the speaker.

- The **overall volume while speaking** = The volume level for each second a participant was categorized as speaking averaged across a shift segment

**S7. Volume while speaking by location:** The overall volume while speaking variable was crossed with the four high level location categories to create four volume by location measures.

- The **volume while speaking in patient rooms** = the average volume while speaking when a participant is located in a patient room for a shift segment
- The **volume while speaking at nursing stations** = the average volume while speaking when a participant is located at a nursing station for a shift segment
- The **volume while speaking in service areas** = the average volume while speaking when a participant is located in a service area for a shift segment
- The **volume while speaking outside of main work areas** = the average volume while speaking when a participant is located outside of main work areas for a shift segment

**POSTURE (P)**

**P1. Overall posture:** Posture data were extracted from Sociometric Solutions, Inc. software. Posture data are derived from the 3 axis accelerometer. Specifically, posture was defined as the overall angular velocity of a worn sensor badge. Posture was sampled at 20 second intervals.

- The **overall posture activity** = average posture across an entire shift segment

**P2. Posture in locations:** Overall posture activity scores were crossed with the four high level location categories to create four posture by location measures.

- The **posture activity in patient rooms** = the average posture when a participant is located in a patient room for a shift segment
- The **posture activity at nursing stations** = the average posture when a participant is located at a nursing station for a shift segment
- The **posture activity in service areas** = the average posture when a participant is located in a service area for a shift segment
- The **posture activity outside main work areas** = the average posture while speaking when a participant is located outside of main work areas for a shift segment

**WALKING (W)**

Walking data were extracted from Sociometric Solutions, Inc. software and based on the activity measure described in the accelerometer-based measures. Specifically, the activity score a threshold value of great than or equal to 0.14 is used to identify segments of walking. An activity score of less than 0.14 was categorized as not walking. For each each second in the 20 second time window, participants were categorized as walking or not.

**W1. Total time walking**

- The **total time walking** = the total number of seconds across an entire shift segment where a participant was classified as walking.
- The **mean time walking** = the total number of seconds spent walking per time window (20 seconds in this analysis) divided by the total number of time windows in the shift segment.

**W2. Time walking in location:** The total time walking variable was crossed with the four high level location categories to create four walking by location measures.

- The **mean time walking in patient rooms** = the average time spent walking when a participant is located in a patient room for a shift segment
- The **mean time walking at nursing stations** = the average time spent walking when a participant is located at a nursing station for a shift segment
- The **mean time walking in service areas** = the average time spent walking when a participant is located in a service area for a shift segment
- The **mean time walking outside main work areas** = the average time spent walking when a participant is located outside of main work areas for a shift segment

**W3. Burstiness of walking:** Two measures of ‘burstiness’ were used to characterize the temporal distribution of walking activity. These were calculated using the same methods described for burstiness of transitions above.

- The **burstiness of walking (FF)** = The Fano Factor of walking
- The **burstiness of walking (CV)** = The coefficient of variation for walking

**TEMPERATURE (T)**

While temperature levels on the ICU were not anticipated to vary considerably, temperature measures were included in the analysis because of the well documented relationship between heat stress and performance.[3] Temperature data were extracted from Sociometric Solutions, Inc. software, sampled at 20 second intervals.

**T1. Overall temperature:** The overall temperature variable was crossed with the four high level location categories to create four temperature by location measures.

- The **overall temperature** = The average temperature level across an entire shift segment

**T2. Temperature in location:** The overall temperature variable was crossed with the four high level location categories to create four temperature by location measures.

- The **temperature in patient rooms** = the average temperature when a participant is located in a patient room for a shift segment
- The **temperature at nursing stations** = the average temperature when a participant is located at a nursing station for a shift segment
- The **temperature in service areas** = the average temperature when a participant is located in a service area for a shift segment
- The **temperature outside main work areas** = the average temperature when a participant is located outside of main work areas for a shift segment

**Supplementary Methods B: Nursing Task Demand Workload Variables**

Nursing task demand measures were captured at the end of each nursing shift. They are organized into three categories: staffing factors (3 variables), patient factors (8 variables), and composite measures (4 variables).

Staffing factors include measures of nurse patient assignments as well as the availability of support staff during a given shift. Patient factors are specific clinical issues associated with each patient a nurse is responsible for during a given shift. These clinical issues were chosen for inclusion by nurses working on the unit where the study took place. Composite measures combine information from staffing and patient factors into summary scores of patient task demands.

**Staffing factors**

- **Number of Patients** = The total number of patients a nurse is directly responsible for during a given shift
- **Number of Patients** **with an Observer** = For any given nursing shift, the total number of observers, also called sitters, assigned to a nurse’s patients. The observer role is intended to support the nurse by monitoring patients continuously, particularly those patients at high risk of fall injury.
- **Nursing Technician / Assistant** = A binary variable indicating whether or not a nurse had an assigned technician or nursing assistant during a given shift. Nursing technician or assistant roles support nurses by completing certain patient care tasks.

**Patient factors**

- **Number of Patients on Insulin Drip** = The total number of patients on an insulin infusion protocol that a nurse is responsible for during a given shift
- **Number of Patients on a Ventilator** = The total number of patients on a ventilator that a nurse is responsible for during a given shift
- **Number of Patients in Isolation** = The total number of patients on an isolation protocol that a nurse is responsible for during a given shift
- **Number of Vasoactive Patients** = The total number of patients receiving blood pressure medication that a nurse is responsible for during a given shift
- **Number of Patients with Flap Checks** = The total number of patients requiring surgical flap checks that a nurse is responsible for during a given shift
- **Number of Patients with Spinal Checks** = The total number of patients requiring spinal flap checks that a nurse is responsible for during a given shift
- **Number of Patients with a PA Catheter** = The total number of patients having a pulmonary artery catheter that a nurse is responsible for during a given shift
- **Number of Patients with CVVHD** = The total number of patients on continuous veno-venous hemodialysis that a nurse is responsible for during a given shift

**Composite measures**

- **Total Patient Factors** = The total number patient factors, a sum of all patient factor variables for the patients a nurse is directly responsible for during a given shift.
- **Average Patient Factors** = The *total patient factors* divided by the *number of patients*
- **Total Patient Load** = A sum of patient status levels over all patients a nurse is directly responsible for during a given shift. There were four levels of patient stats associated with increasing levels of monitoring and intensity of work. Floor status was the lowest (coded as a 1), followed by Intermediate Care (IMC; coded as a two), Progressive Care Unit (PCU; coded as a three), and Intensive Care Unit (ICU; coded as a four) statuses. These statuses were determined by attending physician and nursing leaders at the beginning of each shift.
- **Average Patient Load** = *Total patient load* divided by the *number of patients*

**Supplementary Methods C: Multi-level Modeling Methods**

Level 1 variables included sensor-based measures as predictors, and perceived exertion ratings as dependent variables. Level 1 variables were analyzed in four-hour time blocks or segments within a 12- (or 8-) hour nursing shift. Therefore, Level 1 variables were grouped within the shift in which they were sampled. Level 2 predictors were associated with the shift as a whole and included traditional task demand measures of nursing workload such as patient characteristics and whether or not there was a nursing technician assigned to them during that given shift. Given this data structure, MLM was chosen for two reasons. First, it allows us to account for non-independence in the data collected in multiple shift segments within the same shift. Shift served as the grouping variable in our analysis with each set of sensor-based measurements and self-report ratings from four-hour shift segments nested within the larger shift which usually lasted 12, but sometimes 8 hours. Second, MLM provides a means to test cross-level interactions between task demand variables and sensor-based measures of nursing work processes implied but not yet evaluated empirically by existing frameworks. MLM was conducted in R v. 3.2 using the nlme (v. 3.1-122)[4] and multilevel (v. 2.5)[5] packages.

MLM proceeded in five steps for each dependent variable, mental exertion (ME) and physical exertion (PE). First, ‘intercept’ only mixed-effect models (ME_0_, PE_0_) evaluated the degree to which the shift variable provided a valid grouping structure. Intercept only models were assessed using the following criteria: 1) within group (shift) reliability of means, which by convention should be greater than 0.7,[6] 2) the Intra-class Correlation Coefficient (ICC) describing the proportion of variance between groups (between different shifts) relative to within groups (within a given shift), and 3) the significance of improvement in model fit (reduction in model deviance, AIC) for Models ME_0_ and PE_0_ compared to models that do not include shift as the grouping variable. Second, models including Level 1 Fixed Effect sensor-based measure predictors (ME_1_, PE_1_) were evaluated. Estimation began with the set of individual predictors and interaction terms retained for each dependent variable in the feature selection analysis. All candidate variables were entered and a backward elimination process removed the variable with the largest p-value at each step until only significant variables remained. Non-significant main effect terms for significant interaction terms were retained by convention. Models ME_1_ and PE_1_ were evaluated by the proportion of between and within shift variances accounted for and the improvement in model fit relative to Models ME_0_ and PE_0_. Full Maximum Likelihood estimation was used to allow for significance testing of the improvement in model fit at each step of this process using a Likelihood Ratio (L Ratio) test. Third, models including Level 2 Fixed Effects task demand variables were estimated (ME_2_, PE_2_). This followed the backward elimination and model evaluation processes detailed above. Fourth, models with random coefficients for Level 1 predictors (i.e., variation in slopes between sensor-based measures and perceptions of exertion across shifts) were estimated (ME_3_, PE_3_). Here, random coefficient terms were entered for Level 1 predictors and model fit was evaluated relative to models without random coefficients (ME_2_, PE_2_). Random coefficients for all Level 1 predictors retained after feature selection were considered as it is possible for a predictor to have significant slope variation across a grouping variable without having a significant main effect term.[7] Fifth, cross level interactions between Level 1 and Level 2 variables were explored to account for variation in slopes across shifts (ME_4_, PE_4_). Significance in model fit for Models ME_4_ and PE_4_ were tested relative to ME_3_ and PE_3_. Proportion of variance explained for Level 1 (within shifts) and Level 2 (between shifts) were calculated relative to the ME_0_ and PE_0_ baseline models, and proportion of slope variation explained relative to ME_3_ and PE_3_, the models with random coefficients but no cross-level interactions.

**Supplementary Methods D: Data Dictionary**

This supplement links each measure (described in supplements 1 and 2) with variables in the included data file. Additionally, we indicate whether or not the measure was included in the models reported in this paper. For each outcome measure (perceived ME or PE) we indicate if it was included in the set of measures retained after Elastic Net analysis and whether or not it was included in the final Multi-level Model. Note that in the Elastic Net column Y_x_ indicates that the variable was retained from the pairwise evaluation of interaction terms.

| **Measure Category** | **Measure** | **Variable Name (in data file)** | **Inclusion in reported models** | | | |
| --- | --- | --- | --- | --- | --- | --- |
|  |  |  | **ME** | | **PE** | |
|  |  |  | Elastic Net | Final MLM | Elastic Net | Final MLM |
| *L1. Time in location category* | average time in patient rooms | prop_PR |  |  |  |  |
|  | average time at nursing stations | prop_NS |  |  |  |  |
|  | average time in service areas | prop_Serv |  |  |  |  |
|  | average time outside main work areas | prop_NoLoc | Y_x_ | Y |  |  |
|  | total time in patient rooms | num_PR |  |  |  |  |
|  | total time at nursing stations | num_NS |  |  | Y_x_ | Y |
|  | total time in service areas | num_Serv | Y |  |  |  |
|  | total time outside main work areas | num_NoLoc |  |  |  |  |
| *L2. Movement through physical space* | number of transitions | num_trans |  |  |  |  |
|  | entropy of transitions | entropy | Y |  | Y | Y |
|  | burstiness of transitions (FF) | fano_factor |  |  | Y |  |
|  | burstiness of transitions (CV) | coef_var |  |  | Y_x_ | Y |
| *AI. Overall body movement and activity* | overall body movement | acc_BM_m |  |  |  |  |
|  | overall activity | acc_ACT_m | Y_x_ |  |  |  |
| *A2. Accelerometer-based measures by location* | body movement in patient rooms | PR_acc_BM_m |  |  |  |  |
|  | activity in patient rooms | PR_acc_ACT_m |  |  |  |  |
|  | body movement at nursing stations | NS_acc_BM_m |  |  |  |  |
|  | activity at nursing stations | NS_acc_ACT_m |  |  | Y |  |
|  | body movement in service areas | Serv_acc_BM_m |  |  |  |  |
|  | activity in service areas | Serv_acc_ACT_m |  |  |  |  |
|  | body movement outside main work areas | NoLoc_acc_BM_m |  |  |  |  |
|  | activity outside main work areas | NoLoc_acc_ACT_m | Y_x_ | Y |  |  |
| *E1. Environmental Noise Overall* | overall environmental noise (FV) | aud_FV_m |  |  |  |  |
|  | overall environmental noise (BV) | aud_BV_m |  |  | Y |  |
| *E2. Environmental Noise by location* | environmental noise (FV) in patient rooms | PR_aud_FV_m |  |  |  |  |
|  | environmental noise (BV) in patient rooms | PR_aud_BV_m | Y | Y | Y |  |
|  | environmental noise (FV) at nursing stations | NS_aud_FV_m |  |  |  |  |
|  | environmental noise (BV) at nursing stations | NS_aud_BV_m | Y |  | Y | Y |
|  | environmental noise (FV) in service areas | Serv_aud_FV_m |  |  |  |  |
|  | environmental noise (BV) in service areas | Serv_aud_BV_m | Y_x_ |  | Y_x_ |  |
|  | environmental noise (FV) outside of main work areas | NoLoc_aud_FV_m |  |  |  |  |
|  | environmental noise (BV) outside of main work areas | NoLoc_aud_BV_m |  |  |  |  |
| *S1. Time speaking overall* | total time speaking | t_speak_tot |  |  |  |  |
|  | mean time speaking | t_speak_m |  |  |  |  |
| *S2. Time speaking by location* | total time speaking in patient room | t_speak_PR_tot |  |  | Y |  |
|  | mean time speaking in patient room | t_speak_PR_m |  |  |  |  |
|  | total time speaking at nursing station | t_speak_NS_tot |  |  |  |  |
|  | mean time speaking at nursing station | t_speak_NS_m |  |  |  |  |
|  | total time speaking in service areas | t_speak_Serv_tot |  |  |  |  |
|  | mean time speaking in service areas | t_speak_Serv_m | Y |  |  |  |
|  | total time speaking outside main work areas | t_speak_NoLoc_tot | Y |  | Y | Y |
|  | mean time speaking outside main work areas | t_speak_NoLoc_m | Y |  |  |  |
| *S3. Burstiness of speaking* | burstiness of speaking (FF) | t_speak_ff | Y | Y |  |  |
|  | burstiness of speaking (CV) | t_speak_cv |  |  |  |  |
| *S4. Pitch while speaking overall* | overall pitch while speaking | Pitch_F_wspeak_m |  |  |  |  |
| *S5. Pitch while speaking by location* | pitch while speaking in patient rooms | Pitch_z_F_PR_m | Y |  |  |  |
|  | pitch while speaking at nursing stations | Pitch_z_F_NS_m |  |  |  |  |
|  | pitch while speaking in service areas | Pitch_z_F_Serv_m | Y |  |  |  |
|  | pitch while speaking outside main work areas | Pitch_z_F_NoLoc_m | Y |  |  |  |
| *S6. Volume while speaking overall* | overall volume while speaking | Vol_F_wspeak_m |  |  |  |  |
| *S7. Volume while speaking by location* | volume while speaking in patient rooms | Vol_z_F_PR_m | Y |  |  |  |
|  | volume while speaking at nursing stations | Vol_z_F_NS_m |  |  | Y | Y |
|  | volume while speaking in service areas | Vol_z_F_Serv_m |  |  |  |  |
|  | volume while speaking outside of main work areas | Vol_z_F_NoLoc_m |  |  |  |  |
| *P1. Overall posture* | overall posture activity | Post_act_m |  |  |  |  |
| *P2. Posture in locations* | posture activity in patient rooms | Post_act_PR_m |  |  |  |  |
|  | posture activity at nursing stations | Post_act_NS_m | Y |  |  |  |
|  | posture activity in service areas | Post_act_Serv_m | Y_x_ |  |  |  |
|  | posture activity outside of main work areas | Post_act_NoLoc_m | Y |  |  |  |
| *W1. Total time walking* | total time walking | t_walk_tot |  |  |  |  |
|  | mean time walking | t_walk_m |  |  |  |  |
| *W2. Time walking in location* | mean time walking in patient rooms | t_walk_PR_m | Y |  | Y_x_ | Y |
|  | mean time walking at nursing stations | t_walk_NS_m |  |  |  |  |
|  | mean time walking in service areas | t_walk_Serv_m | Y |  |  |  |
|  | mean time walking outside main work areas | t_walk_NoLoc_m |  |  |  |  |
| *W3. Burstiness of walking* | burstiness of walking (FF) | t_walk_ff | Y |  |  |  |
|  | burstiness of walking (CV) | t_walk_cv |  |  |  |  |
| *T1. Overall temperature* | overall temperature | Temperature_m |  |  |  |  |
| *T2. Temperature in location* | temperature in patient rooms | Temperature_PR_m |  |  |  |  |
|  | temperature at nursing stations | Temperature_NS_m |  |  |  |  |
|  | temperature in service areas | Temperature_Serv_m | Y |  | Y_x_ | Y |
|  | temperature outside of main work areas | Temperature_NoLoc_m |  |  |  |  |
| *Staffing factors* | Number of patients | Number_Pts |  |  |  |  |
|  | Number of patients with an Observer | Tot_Observer |  |  |  |  |
|  | Nursing technician / assistant | Tech_Y_N |  |  |  |  |
| *Patient factors* | Number of patients on Insulin Drip | Tot_Insulin_drip |  | Y |  |  |
|  | Number of patients on a Ventilator | Tot_Ventilator |  |  |  |  |
|  | Number of patients in Isolation | Tot_Isolation |  |  |  |  |
|  | Number of Vasoactive patients | Tot_Vasoactive |  |  |  |  |
|  | Number of patients with flap checks | Tot_Checks_Flap |  |  |  |  |
|  | Number of patients with spinal checks | Removed (no patients with this factor included in data) |  |  |  |  |
|  | Number of patients with a PA catheter | Removed (no patients with this factor included in data) |  |  |  |  |
|  | Number of patients with CVVHD | Removed (no patients with this factor included in data) |  |  |  |  |
| *Composite measures* | Total patient factors | Total_Pt_Factors |  |  |  |  |
|  | Average patient factors | AVG_Pt_Factors_G |  |  |  |  |
|  | Total Patient Load | Pt_load |  |  |  |  |
|  | Average Patient Load | AVG_Pt_Load_G |  |  |  | Y |
| *Self-report exertion* | Physical exertion | Physical_ex |  |  |  |  |
|  | Mental exertion | Mental_ex |  |  |  |  |
| *Identifiers* | Unique identifier for each shift | Shift_number |  |  |  |  |
|  | Time block within shift | TimeBlock |  |  |  |  |
|  | Starting hour of day for time block | Hour_of_day |  |  |  |  |
|  | Day of week for shift | Day_of_week |  |  |  |  |

**References:**

1. Szalma JL, Hancock PA. Noise effects on human performance: a meta-analytic synthesis. *Psychol Bull*. 2011;137(4):682-707. doi:10.1037/a0023987.
2. Olguín-Olguín, D. & Pentland, A. Sensor-based organisational design and engineering. *Int. J. Organ. Des. Eng.* **1,** 69–97 (2010).
3. Hancock PA, Ross JM, Szalma JL. A meta-analysis of performance response under thermal stressors. Human Factors: The Journal of the Human Factors and Ergonomics Society. 2007 Oct 1;49(5):851-77.
4. R Core Team (2015). R: A language and environment for statistical computing. R Foundation for Statistical Computing, Vienna, Austria. URL <https://www.R-project.org/>.
5. Pinheiro J, Bates D, DebRoy S, Sarkar D and R Core Team (2016). *nlme: Linear and Nonlinear Mixed Effects Models*. R package version 3.1-126, [http://CRAN.R-project.org/package=nlme](http://cran.r-project.org/package=nlme).
6. Bliese, P. D. in Multilevel theory, research, and methods in organizations: Foundations, extensions, and new directions (eds. Klein, K. J. & Kozlowski, S. W. J.) 349–381 (Jossey-Bass, 2000).
7. Hox, J. J., Moerbeek, M. & Schoot, R. van de. Multilevel Analysis: Techniques and Applications, Second Edition. (Routledge, 2010).
